# Prevalence of Comorbidities in Inflammatory Bowel Disease: An Umbrella Review of 18 Systematic Reviews

**DOI:** 10.3390/jcm15051739

**Published:** 2026-02-25

**Authors:** Lupita Ana Maria Valladolid-Sandoval, Jhosmer Ballena-Caicedo, Fiorella E. Zuzunaga-Montoya, Darwin A. León-Figueroa, Percy Ordemar Vásquez, Mario J. Valladares-Garrido, Víctor Juan Vera-Ponce

**Affiliations:** 1 Facultad de Medicina (FAMED), Universidad Nacional Toribio Rodríguez de Mendoza de Amazonas (UNTRM), Chachapoyas 01001, Amazonas, Peru; luamavasa1911@gmail.com (L.A.M.V.-S.); 7330178022@untrm.edu.pe (J.B.-C.); victor.vera@untrm.edu.pe (V.J.V.-P.); 2 Universidad Continental, Lima 15306, Peru; fiorellazuzunaga@gmail.com; 3 Facultad de Medicina Humana, Universidad de San Martín de Porres, Chiclayo 15011, Peru; darwin_leon@usmp.pe; 4 EpiHealth Research Center for Epidemiology and Public Health, Lima 15102, Peru; 5 Escuela de Medicina Humana, Universidad Señor de Sipán, Chiclayo 14001, Peru; ordemarper@uss.edu.pe

**Keywords:** inflammatory bowel diseases, Crohn Disease, ulcerative colitis, comorbidity, meta-analysis, extraintestinal manifestations, non-alcoholic fatty liver disease

## Abstract

**Background:** Inflammatory bowel disease (IBD) is associated with numerous extraintestinal manifestations and systemic comorbidities; however, the certainty of prevalence estimates across multiple organ systems has not been systematically evaluated. **Objective:** To synthesize evidence from systematic reviews on the prevalence of comorbidities in patients with inflammatory bowel disease (IBD) and to assess the certainty of estimates through an umbrella review. **Methods:** In this umbrella review, we included systematic reviews reporting the prevalence of comorbidities in adults with IBD and their confidence intervals. Methodological quality was assessed using AMSTAR-2 and ROBIS, while statistical heterogeneity and certainty of evidence were evaluated using GRADE adapted for prevalence studies. **Results:** Eighteen systematic reviews published between January 2014 and September 2025 were included. The highest prevalences were sexual dysfunction 50.6% (95% CI 40.8–60.5), fecal incontinence in Crohn’s disease 34.8% (27.9–41.9), non-alcoholic fatty liver disease 32% (24–40), anemia 24% (18–31), and ≥1 extraintestinal manifestation 24% (19–31). Only four comorbidities achieved moderate certainty: primary sclerosing cholangitis 2.16% (1.76–2.60), uveitis 2.38% (1.60–3.17), hepatitis B 3.3% (2.5–4.0), and hepatitis C 1.8% (1.2–2.4). Prevalence rates varied significantly by IBD subtype, geographic region, and diagnostic method. Heterogeneity was consistently high (I^2^ > 90%), and certainty was predominantly low or very low. **Conclusions:** Comorbidities in IBD are frequent, with prevalences ranging from 1.8% to 50.6%, highlighting the importance of comorbidity awareness in clinical practice. However, the certainty of evidence is predominantly low or very low due to extreme methodological heterogeneity. These findings underscore the urgent need for studies with standardized diagnostic methods and robust statistical approaches to strengthen the evidence base and establish evidence-based surveillance protocols.

## 1. Introduction

Inflammatory bowel disease (IBD)—ulcerative colitis and Crohn’s disease—represents a growing global health burden, with prevalence exceeding that of rare diseases and continuing to rise according to the Global Burden of Disease [1]. The epidemiological transition described for IBD—with scenarios of emergence, acceleration of incidence, and compounding prevalence—illustrates that while incidence stabilizes in industrialized countries, it continues to grow in newly industrialized regions, anticipating sustained increases in prevalence [2,3].

Beyond intestinal involvement, IBD is characterized by a broad spectrum of extraintestinal manifestations and systemic comorbidities that compromise multiple organs and systems. Extraintestinal manifestations (EIMs) include articular involvement (axial and peripheral spondyloarthritis), dermatological (erythema nodosum, pyoderma gangrenosum), ocular (uveitis, episcleritis), and hepatobiliary (primary sclerosing cholangitis) manifestations, among others. These EIMs may precede intestinal diagnosis, accompany disease flares, or follow an independent course, and contribute substantially to disability, impaired quality of life, and therapeutic complexity. Additionally, patients with IBD present increased risk of metabolic comorbidities (hepatic steatosis, metabolic syndrome), infectious (bacterial overgrowth, opportunistic pathogen infections), bone-related (osteoporosis), and functional (sexual dysfunction, fecal incontinence) complications, whose recognition and integration into clinical management remain suboptimal. ECCO guidelines and landmark reviews have consolidated the recognition of EIMs as an integral part of the “systemic phenotype” of IBD [4,5].

We focused specifically on prevalence estimates rather than incidence rates or measures of association (relative risks, odds ratios) for several methodological and clinical reasons. Prevalence data directly inform clinical practice by answering the question of what proportion of IBD patients have a given comorbidity at any point in time, which is essential for planning screening strategies and resource allocation. In contrast, incidence studies require longitudinal cohort designs with defined follow-up periods, while association studies require appropriate comparison groups—both representing fundamentally different research questions that would be methodologically inappropriate to combine with prevalence data in a single synthesis.

Although multiple systematic reviews have documented specific comorbidities in IBD, the evidence remains fragmented across organs and disciplines, with heterogeneous diagnostic definitions and diverse population frameworks. This proliferation of individual systematic reviews, combined with the absence of a comprehensive quality assessment across the field, makes an umbrella review—rather than another systematic review of primary studies—the most appropriate methodological approach to synthesize existing evidence and evaluate its certainty. Critically, no previous synthesis has systematically assessed the certainty of these prevalence estimates, limiting their applicability for clinical decisions regarding surveillance and screening. Therefore, we conducted an umbrella review with two objectives: (1) to quantitatively synthesize reported prevalences of comorbidities in IBD; and (2) to assess the certainty of these estimates using an adapted GRADE framework, identifying the main sources of methodological heterogeneity (diagnostic methods, population characteristics, geographic distribution) to inform clinical practice recommendations stratified by level of evidence.

## 2. Materials and Methods

### 2.1. Study Design

We conducted an umbrella review focused on estimating and comparing the prevalence of comorbidities in patients with inflammatory bowel disease (IBD). The conduct and reporting followed the Preferred Reporting Items for Overviews of Reviews (PRIOR) guidelines for overviews [6], the Preferred Reporting Items for Systematic Reviews and Meta-Analyses (PRISMA) guidelines (Table S1), and the Joanna Briggs Institute (JBI) methodological framework for synthesis of reviews [7].

### 2.2. Search Strategy

A comprehensive search was performed in MEDLINE/PubMed, Embase, Scopus, and Web of Science—Core Collection, from inception to the cutoff date (5 September 2025), without country and language restrictions. Controlled and free-text terms related to IBD, prevalence, and systematic review designs were combined: (“inflammatory bowel disease” OR “Crohn*” OR “ulcerative colitis”) AND (“prevalence” OR “proportion”) AND (“systematic review” OR “meta-analysis”). The search strategy was developed in PubMed by two independent reviewers (VJVP and JJBC) and subsequently adapted for each additional database. Any discrepancies in the search strategy development were resolved through discussion between these two reviewers. This adaptation can be examined in detail in Table S2.

### 2.3. Selection Criteria

We included systematic reviews with or without meta-analysis that had as their primary objective to determine the prevalence of comorbidities in adults with a diagnosis of IBD (Crohn’s disease, ulcerative colitis, or IBD unclassified). Studies must report prevalence estimates with confidence intervals and provide explicit definitions of both IBD and the comorbidity evaluated.

We excluded: (1) narrative reviews, clinical guidelines, editorials, and letters to the editor; (2) single-center studies without systematic synthesis; (3) reviews that only reported measures of association (odds ratio, relative risk) without estimating prevalences; (4) studies focused exclusively on risk factors or predictors without quantifying the frequency of the comorbidity; and (5) reviews that evaluated prevalence of IBD in populations with specific comorbidities (inverse direction of association).

### 2.4. Definition of IBD and Comorbidities

We accepted the IBD definitions used by each included review, recognizing the diversity of diagnostic criteria employed in clinical practice (clinical-endoscopic and histological diagnosis, International Classification of Diseases [ICD] codes, or population registries) [8,9].

For each comorbidity, we systematically documented the diagnostic method used, categorizing them according to validity hierarchy: (1) histological or radiological confirmation (primary sclerosing cholangitis [PSC] by MRCP/ERCP, uveitis by ophthalmologist); (2) validated clinical criteria (spondyloarthritis by ASAS/modified NY); (3) standardized serum biomarkers (hepatitis by HBsAg/anti-HCV); (4) validated questionnaires (sexual dysfunction by FSFI/IIEF); or (5) administrative codes (ICD-9/10). This stratification allowed assessment of the impact of diagnostic method on prevalence estimates in the risk of bias domain of the GRADE framework.

### 2.5. Data Extraction

Three reviewers (LAMVS, JJBC and FEZM) independently extracted the following data using standardized forms: study characteristics (first author, year of publication, geographic region, search period), study population (total sample size and by IBD subgroup), diagnostic methodology (operational definition of IBD and method of comorbidity confirmation), primary outcomes (number of cases, percentage prevalence with 95% CI), meta-analysis characteristics when applicable (heterogeneity statistic I^2^, synthesis model used, subgroup analyses by IBD type, geographic region, sex, or age), and methodological quality (declared risk of bias assessment tool and main limitations identified by the authors).

When a review provided multiple stratified estimates (by diagnostic method, geographic region, or population), all available estimates were extracted to facilitate sensitivity analyses and exploration of sources of heterogeneity. Discrepancies in extraction were resolved through discussion among reviewers or, when necessary, consultation with a fourth reviewer (VJVP).

### 2.6. Risk of Bias Assessment of Reviews

AMSTAR-2 [10] was applied (16 items; critical domains: a priori protocol, comprehensive search strategy, justification for exclusions, risk of bias assessment of primary studies, appropriate meta-analysis methods, consideration of risk of bias in interpretation, and publication bias assessment) to rate each review as high, moderate, low, or critically low confidence. In parallel, ROBIS (12) was applied (Phase 2: eligibility criteria, identification and selection of studies, data collection and appraisal, and synthesis and findings) to determine overall risk of bias (low, high, or unclear). Risk of bias assessment was performed independently by three reviewers (LAMVS, JJBC, and FEZM). Disagreements were resolved through discussion or consultation with a fourth reviewer (VJVP) when necessary.

### 2.7. Synthesis and Selection of the Most Appropriate Estimate

For each comorbidity with multiple systematic reviews available, we selected a single main estimate using a predefined hierarchical algorithm: (1) lowest methodological risk of bias (low ROBIS + AMSTAR-2 with ≥3/7 critical domains met); (2) greater coverage (number of studies and sample size); (3) lower statistical heterogeneity (I^2^); and (4) most rigorous diagnostic method (clinical/radiological confirmation over administrative codes). Supplementary Material Table S3 documents all reviews evaluated per comorbidity and justifies each prioritization decision. Overlap of primary studies across systematic reviews is an inherent challenge in umbrella reviews. To address this, we implemented two strategies: (1) we selected a single systematic review per comorbidity using predefined hierarchical criteria, thereby avoiding the combination of potentially overlapping estimates; and (2) we did not recalculate meta-analyses or perform meta-meta-analyses, which would amplify unit-of-analysis errors due to overlap of primary studies [11]. Table S3 documents all reviews evaluated per comorbidity, specifying which were selected and the rationale for each decision.

### 2.8. Assessment of the Certainty of Evidence

To contextualize confidence in reported prevalence estimates, we applied an adapted framework from the GRADE system (Grading of Recommendations Assessment, Development and Evaluation) for observational frequency studies, following the Joanna Briggs Institute guidelines for synthesis of evidence on prevalence and incidence [12]. Although GRADE was originally developed to evaluate therapeutic interventions [13], its application to prevalence studies can be implemented through adaptation of its five core domains [14]: (1) risk of bias, assessing sampling methods, population representativeness, and validation of diagnostic definitions; (2) inconsistency, considering statistical heterogeneity (I^2^), the breadth of prevalence ranges across primary studies, and the degree of explanation through subgroup analyses; (3) indirectness, evaluating differences between studied populations and the target population (adults with confirmed IBD), including obsolescence of diagnostic definitions and representativeness of healthcare settings; (4) imprecision, examining the width of 95% confidence intervals relative to the point prevalence estimate and total sample size; and (5) publication bias, when formally assessed through statistical tests (funnel plot, Egger/Begg test) in included systematic reviews.

The certainty of evidence was classified into four categories: high (very confident that the estimate is close to the true value), moderate (moderate confidence; the true value is probably close but may differ substantially), low (limited confidence; the true value may be substantially different), or very low (very little confidence; the true value is almost certainly substantially different). Each domain could downgrade certainty by one or two levels, depending on the severity of identified limitations. The assessment was performed independently by three reviewers (LAMVS, JJBC, and FEZM) for each included comorbidity, with discrepancies resolved by consensus or consultation with a fourth reviewer (VJVP). Since we evaluated systematic reviews rather than individual primary studies, we integrated the methodological quality information already obtained through AMSTAR-2 and ROBIS into the assessment of the “risk of bias” domain, complementing it with the specific evaluation of methodological heterogeneity in diagnostic methods and studied populations reported by each review. This approach allows interpretation of prevalence estimates with explicit knowledge of their level of uncertainty, facilitating informed decisions about their clinical applicability and additional research needs.

### 2.9. Ethical Considerations

This work uses published aggregate data and does not involve identifiable individual information; therefore, it does not require ethical approval. Good practices of transparency in evidence synthesis were respected (a priori protocol and availability of Supplementary Materials).

## 3. Results

### 3.1. Article Selection Process

The systematic search identified 14,124 records across the four aforementioned databases, with no additional records from other sources. After removing 5680 duplicates, 8444 records were screened by title and abstract, excluding 8399 primarily because they were not systematic reviews or meta-analyses (n = 1746), did not report prevalence of comorbidities (n = 6353), had non-IBD or mixed populations without stratification (n = 1111), and duplicates not automatically detected (n = 189). Forty-five full-text articles were evaluated, with 27 excluded for various methodological reasons. Finally, 18 systematic reviews were included in the qualitative synthesis (Figure S1) [15,16,17,18,19,20,21,22,23,24,25,26,27,28,29,30,31,32].

It is important to note that during the selection process, six additional systematic reviews were identified that shared the same objective (evaluating the prevalence of the same comorbidity) as already included studies. In these cases, a single review per comorbidity was selected following predefined prioritization criteria. Table S3 presents a detailed table with these reviews of shared objectives, specifying which was selected as the main estimate and the specific reasons for each decision.

### 3.2. Study Characteristics

The set of included reviews encompasses publications between 2014 and 2025, with predominance of adult populations and highly variable sample sizes (Table 1). For example, the systematic review on non-alcoholic fatty liver disease (NAFLD) integrated 27 studies and 7640 patients, with a median age of approximately 41 years and a sex distribution close to 50% when reported; additionally, it combined prospective and retrospective studies, and a substantial portion came from conference abstracts, which explains part of the heterogeneity between estimates [16]. In Clostridioides difficile infection (CDI), the recent systematic review integrated 796,244 patients from 28 studies; the median age was 39 years (interquartile range [IQR] 36–43), and 47% were male, with a wide range of sample sizes from 54 to 562,798 individuals [17]. For anemia, the European systematic review based on individual patient data included 2192 outpatient adults and reported a mean age of 43.9 years [21]. The frailty systematic review analyzed nine studies with 1,495,695 patients with IBD, using multiple validated scales (Hospital Frailty Risk Score, Clinical Frailty Index, Survey of Health Frailty Index, modified Frailty Index), with a pooled prevalence of 18% and a significant association with mortality and major adverse events [24].

Geographically, the evidence is well represented in Europe, North America, and Asia, with more specific appearances in South America and Africa depending on the comorbidity. NAFLD included 12 studies from North America, 11 from Europe, and 4 from Asia [16]. Uveitis included studies from Europe (Switzerland, Denmark, England, Greece, Spain, Italy) and Asia (India, China, Turkey, South Korea, Taiwan), in addition to two regional multicenter studies; all in adults (21). Primary sclerosing cholangitis (PSC) compiled evidence from 30 countries encompassing North America, South America, Europe, the Middle East, and Asia [15]. CDI provided data from 11 countries across three continents (Asia, Europe, and North America) [17]. Other comorbidities show specific coverage: anemia was restricted to Europe (Germany, Greece, Italy, Spain, Switzerland, Denmark, Norway, and Sweden) [21], bronchiectasis included the United States, Japan, Greece, Canada, France, Turkey, the United Kingdom, India, and Poland [26], metabolic syndrome analyzed series from the United States, Serbia, Italy, Portugal, Japan, Korea, and Turkey, with overall moderate methodological quality [27], and the extraintestinal manifestations review covered mainly Europe, Asia, North America, and the Middle East, with 52 studies and over 352,000 patients [25].

### 3.3. Diseases Studied and Diagnostic Methods

Diagnostic methods varied substantially across included reviews, which we categorized hierarchically by validity: (1) histological or radiological confirmation (e.g., PSC by MRCP/ERCP, NAFLD—now termed MASLD following the 2023 multi-society Delphi consensus, though we retain the original terminology as included studies predate this change—by biopsy); (2) validated clinical criteria (e.g., spondyloarthritis by ASAS/modified New York criteria, uveitis by ophthalmological confirmation); (3) standardized biomarkers (e.g., hepatitis by HBsAg/anti-HCV, anemia by WHO/ECCO hemoglobin thresholds); (4) validated questionnaires (e.g., sexual dysfunction by FSFI/IIEF, fecal incontinence by FIQL/CCF-FI, frailty by HFRS/mFI); and (5) administrative codes (ICD-9/10). Detailed diagnostic criteria for each comorbidity are presented in Table 1.

This methodological heterogeneity constitutes the primary source of variability in prevalence estimates and certainty downgrading, as detailed in the Discussion. The impact of diagnostic method on reported frequencies is illustrated by substantial differences observed across comorbidities (e.g., NAFLD, bronchiectasis, *C. difficile* infection), where more sensitive techniques consistently yielded higher prevalence estimates.

### 3.4. Age Distribution and Age Groups

Globally, the evidence is predominantly in young to middle-aged adults. NAFLD and uveitis excluded pediatric populations and concentrated mean ages in the fourth and fifth decades, with a median of approximately 41 years in NAFLD and similar ranges in uveitis [16,19]. In CDI, the median age of 39 years (IQR 36–43) and 47% male describe a sample also centered on adults, with a tendency toward higher incidence in males and with age, according to meta-regression [17].

Pediatric representation is limited and appears more visibly in dermatology/ophthalmology. In the systematic review of psoriasis and psoriatic arthritis, higher prevalences are reported in children/adolescents with IBD than in adults (e.g., psoriasis 8.4% in <18 years versus 3.8% in ≥18 years), although the pediatric study base is small and heterogeneous (20). In anemia, although the European systematic review included only outpatient adults, it reported estimates by age strata (18–29, 30–39, 40–49, 50–64, 65–74, and >74 years), with moderate variations between groups and a clear weight of IBD activity on prevalence [21].

### 3.5. Main Prevalences

**Figure 1:**
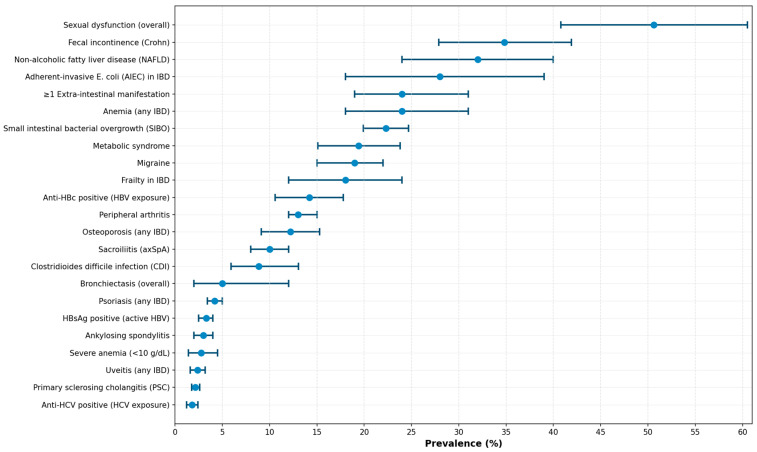
Pooled prevalences of comorbidities in inflammatory bowel disease.

Pooled prevalences show a heterogeneous profile across comorbidities and regions (Table 1 and Figure 1). All estimates presented very high statistical heterogeneity (I^2^ >80–90%), reflecting substantial variability between primary studies attributable to differences in diagnostic methods, studied populations (outpatient vs. hospitalized; population-based vs. tertiary series), and geographic regions. Therefore, pooled prevalences should be interpreted as approximate estimates of the expected range rather than precise values universally applicable to all clinical contexts.

NAFLD showed the highest prevalence: 32% (95% confidence interval [95% CI] 24–40), with marked geographic variation (North America 43%, Europe 31%, Asia 13%); advanced fibrosis in IBD reached 10.3% [16]. PSC presented a prevalence of 2.16% in overall IBD (UC 2.47%; CD 0.96%), with variation by diagnostic method and region [15].

At least one extraintestinal manifestation (EIM) was documented in 24% (95% CI 19–31) of patients with IBD, with prevalences of 27% in UC and 35% in CD [25]. This global prevalence encompasses articular, dermatological, ocular, and hepatobiliary manifestations, whose specific frequencies are detailed below. The specific prevalences of individual extraintestinal manifestations are not additive, as some patients present multiple simultaneous EIMs; therefore, the sum of specific prevalences may exceed the global prevalence of ≥1 EIM due to overlap between conditions.

Psoriasis occurred in 4.2% (95% CI 3.4–5.0) of patients with IBD, with 3.6% in CD and 2.8% in UC, and higher prevalences in the pediatric population (8.4% in <18 years vs. 3.8% in ≥18 years). Psoriatic arthritis affected approximately 1.0% (95% CI 0–1.9) of patients with IBD [18]. Uveitis showed a pooled prevalence of 2.38% (95% CI 1.60–3.17) in IBD, with significant differences by type (CD 3.27%; UC 1.60%) and geographic region (lower frequency in Asia than in Europe) [19]. For spondyloarthritis: sacroiliitis 10% (8–12%), ankylosing spondylitis 3% (2–4%), and peripheral arthritis 13% (12–15%), with wide differences by diagnostic criteria and settings [20]. Bronchiectasis presented an overall prevalence of 5% (2–12%), rising to 12% (4–39%) in studies that systematically applied HRCT, suggesting underdiagnosis with less sensitive methods [26].

SIBO affected 22.3% of patients with IBD (CD 25.4%; UC 14.3%) [28]. CDI showed an overall prevalence of 8.84% (Asia 11.3%; North America 7.85%; Europe 7.92%), with higher rates when diagnosis was based on PCR versus EIA [17]. AIEC presented a prevalence of 28% in IBD (CD 29%; UC 13%), although based on small samples with heterogeneous methods [22]. For viral markers, positive HBsAg was 3.3% (2.5–4.0%), anti-HBc 14.2% (10.6–17.8%), anti-HCV 1.8% (1.2–2.4%), and HCV-RNA 0.8% (0.4–1.3%), with high geographic heterogeneity [23].

Anemia was documented in 24% of European patients with IBD (CD 27%; UC 21%; severe 2.75%), with iron deficiency in 57% of cases [21]. Metabolic syndrome showed a prevalence of 19.4% (15.1–23.8%), with wide differences by IBD type: UC 38.2% vs. CD 13.6%. This apparent discordance may reflect differences in studied populations, metabolic syndrome definitions employed (ATP-III, IDF, AHA/NHLBI), or differential corticosteroid exposure between cohorts. Sensitivity analysis excluding lower-quality studies yielded a prevalence of 21.9% (18–25.8%) with attenuated heterogeneity [27].

Osteoporosis affected 12.2% (95% CI 9.1–15.3%) of patients with IBD, without significant differences between CD (14.9%) and UC (11.4%), but with a higher prevalence in women (10.5%) compared to men (9.6%), although this difference did not reach statistical significance. Prolonged corticosteroid exposure was associated with a higher prevalence of osteoporosis [29].

Frailty occurred in 18% (95% CI 12–24) of patients with IBD, with significant association with all-cause mortality (pooled hazard ratio approximately 2.25, 95% CI not reported in the systematic review) and major adverse events, with greater frequency in elderly populations and in the presence of active disease or multiple comorbidities [24].

Migraine presented an overall prevalence of 19% (15–22%), with differences by IBD type: UC 10% (4–15%) and CD 24% (17–30%) [30]. Sexual dysfunction affected 50.6% (40.8–60.5%) of patients with IBD, with higher prevalence in women (62.7%) than in men (34.0%) and during periods of disease activity (75.1% vs. 34.2% in remission) [32]. Fecal incontinence in Crohn’s disease reached 34.8% (27.9–41.9%), with very high heterogeneity (I^2^ ≈ 99%) and several significant predictors, including previous surgery. Evidence for ulcerative colitis was insufficient to estimate the pooled prevalence [31].

### 3.6. AMSTAR-2 Assessment

The AMSTAR-2 assessment revealed common methodological limitations that compromise confidence in the estimates (Table S4). The most frequent deficiencies included the absence of a priori registered protocols (only Alinaghi et al., 2020 reported a prior protocol) [18], searches restricted exclusively to the English language in multiple reviews, and non-duplicated or unreported data extraction in several studies. The risk of bias assessment of primary studies showed heterogeneity in approaches, predominantly using the Newcastle-Ottawa Scale (NOS) or AHRQ tools, but without systematic integration of these findings into the interpretation of results.

The most recent reviews (2021–2025) demonstrated better methodological standards, particularly in the implementation of sensitivity analyses and exploration of heterogeneity. For example, Lin et al. [16] stratified NAFLD estimates by diagnostic method and geographic region, while Shen et al. [27] performed a sensitivity analysis that reduced metabolic syndrome heterogeneity from 81% to 52%. However, publication bias assessment was inconsistent, being adequately reported only in the reviews by Shah et al. (2019) [28], Amakye et al. (2025) [17], Alinaghi et al. (2020) [18], Lin et al. (2024) [19], Huang et al. (2022) [24], and Kiliç et al. (2024) [25]. The overall rating resulted in low confidence for most reviews (n = 13), moderate confidence for one review, low-moderate confidence for another, and critically low confidence for two reviews due to multiple deficiencies in critical domains.

### 3.7. ROBIS Assessment

The ROBIS assessment identified concerns concentrated primarily in the synthesis and interpretation domains (Domain 4), where methodological heterogeneity and variability in diagnostic definitions constituted the main limitations (Table S5). Disparate diagnostic definitions were particularly problematic in NAFLD (imaging versus elastography versus biopsy), PSC (administrative codes versus radiological confirmation), and bronchiectasis (radiography versus HRCT), generating estimates that were not directly comparable across primary studies.

Domain 3 (data collection and study appraisal) showed concerns in reviews that combined heterogeneous designs or used administrative codes without adequate clinical verification. Most reviews presented low risk in the eligibility (Domain 1) and identification/selection (Domain 2) domains, reflecting appropriate search strategies and clearly defined inclusion criteria. Overall risk was classified as moderate only for Lin et al. (2024) [19], while the remaining reviews presented high risk or “some concerns” mainly due to unexplained heterogeneity and limitations in methodological reporting. Additionally, persistently high statistical heterogeneity (I^2^ > 80–90% in most analyses) and limited publication bias assessment represent cross-cutting limitations that affect the certainty of evidence, regardless of the individual quality of each systematic review.

### 3.8. Certainty of Evidence for Prevalence Estimates

Assessment of certainty using the adapted GRADE framework revealed that confidence in prevalence estimates was predominantly low or very low for most evaluated comorbidities (Table 2). Only four comorbidities achieved moderate certainty: primary sclerosing cholangitis (2.16%, 95% CI 1.76–2.60), uveitis (2.38%, 1.60–3.17), and exposure to hepatitis B (HBsAg 3.3%, 2.5–4.0) and hepatitis C (anti-HCV 1.8%, 1.2–2.4). These comorbidities shared favorable methodological characteristics: more homogeneous and validated diagnostic definitions (PSC by magnetic resonance cholangiopancreatography or endoscopic retrograde cholangiopancreatography; uveitis with ophthalmological confirmation in 57% of studies; hepatitis by standardized serology), substantial sample sizes (from 10,304 to 776,700 patients), and relatively narrow confidence intervals. However, even these estimates showed high statistical heterogeneity (I^2^ > 75–99%), mainly due to genuine geographic variation in the case of viral hepatitis and differences in ascertainment methods (administrative codes versus clinical-radiological confirmation) in PSC and uveitis, which prevented achieving high certainty for any comorbidity.

Eight comorbidities presented low certainty: anemia (24%, 18–31%), Clostridioides difficile infection (8.84%, 5.91–13.03%), small intestinal bacterial overgrowth (22.3%, 19.9–24.7%), fecal incontinence in Crohn’s disease (34.8%, 27.9–41.9%), metabolic syndrome (19.4%, 15.1–23.8%), osteoporosis (12.2%, 9.1–15.3%), frailty (18%, 12–24%), presence of at least one extraintestinal manifestation (24%, 19–31%), and psoriasis (4.2%, 3.4–5.0%). The main reasons for downgrading were extreme statistical heterogeneity (I^2^ > 90% in all these comorbidities) with only partial explanation through subgroup analyses, methodological variability in diagnostic definitions or measurement instruments (for example, ATP-III versus IDF criteria for metabolic syndrome; multiple non-standardized frailty scales; mixture of dual-energy X-ray absorptiometry and administrative codes for osteoporosis), and limited representativeness with predominance of European and North American tertiary centers.

Six comorbidities obtained very low certainty: non-alcoholic fatty liver disease (32%, 24–40%), axial and peripheral spondyloarthritis (10% and 13% respectively), adherent-invasive *E. coli* (28%, 18–39%), bronchiectasis (5%, 2–12%), migraine (19%, 15–22%), sexual dysfunction (50.6%, 40.8–60.5%), and psoriatic arthritis (1.0%, 0–1.9%). These estimates accumulated severe downgrades in multiple domains simultaneously: very serious risk of bias due to non-standardized diagnostic methods (for example, heterogeneous in vitro assays for adherent-invasive *E. coli*; non-comparable questionnaires for sexual dysfunction), very serious inconsistency with I^2^ > 95% without adequate explanation, indirect evidence from highly selected populations or obsolete definitions (NAFLD pre-MASLD 2023 nomenclature), and very serious imprecision due to small sample sizes and extremely wide confidence intervals (for example, adherent-invasive *E. coli* with only 465 total patients; psoriatic arthritis with CI 0–1.9% that includes the null value).

## 4. Discussion

### 4.1. Main Findings

This umbrella review demonstrates that comorbidities in IBD are frequent across multiple organ systems, with pooled prevalences ranging from 1.8% (hepatitis C) to 50.6% (sexual dysfunction). The highest prevalences were observed for sexual dysfunction (50.6%), fecal incontinence in Crohn’s disease (34.8%), NAFLD (32%), anemia (24%), and presence of at least one extraintestinal manifestation (24%). However, a critical finding that transcends these individual figures is that the certainty of evidence is predominantly low or very low. Of 18 evaluated comorbidities, only four achieved moderate certainty (PSC, uveitis, hepatitis B/C), all characterized by standardized and validated diagnostic methods, while comorbidities with the greatest impact on quality of life presented very low certainty due to extreme methodological heterogeneity (I^2^ > 95%), non-standardized definitions, and small sample sizes.

The impact of the diagnostic method on prevalence estimates constituted the main source of certainty downgrading. This methodological dependence, consistently documented across multiple comorbidities and detailed in the following section, reveals that much of the observed ‘heterogeneity’ does not reflect genuine clinical variation but rather a technical artifact, making pooled estimates potentially misleading in specific contexts.

### 4.2. Comparison with Current Literature

Our findings align with recent comprehensive reviews conceptualizing IBD as a multisystem disorder rather than a purely gastrointestinal condition [5,33]. Rogler et al. reported that extraintestinal manifestations can occur in up to 24% of patients before the onset of intestinal symptoms [5], consistent with our pooled estimate of 24% (95% CI 19–31) for at least one extraintestinal manifestation. The global epidemiological transition of IBD, with compounding prevalence in industrialized regions and accelerating incidence in newly industrialized countries [34], underscores the growing clinical relevance of systematic comorbidity surveillance.

Notably, our umbrella review did not include psychiatric comorbidities, which represent a substantial disease burden in IBD populations. Recent meta-analyses have documented anxiety symptoms in approximately 32% and depression symptoms in 25% of IBD patients, with prevalence rates increasing during active disease [35]. In hospitalized IBD patients, psychiatric comorbidities have shown increasing temporal trends: a United States nationwide analysis from 2009–2018 reported that mood disorders and anxiety diagnoses were associated with longer hospital stays, higher hospitalization costs, and increased 30-day readmission rates [36]. The rising burden of psychiatric and behavioral disorders has also been documented in pediatric IBD populations, with depression, anxiety, and substance use disorders each affecting approximately 22% of hospitalized patients [37]. These findings support the integration of mental health screening into comprehensive IBD care, particularly in hospitalized patients where psychiatric comorbidity may adversely affect clinical outcomes.

Similarly, autoimmune comorbidities were beyond the scope of the systematic reviews included in our analysis but constitute an important component of IBD-associated morbidity. Population-based studies have identified associations between IBD and at least 24 autoimmune conditions, including thyroid disorders, rheumatoid arthritis, type 1 diabetes mellitus, multiple sclerosis, and autoimmune hepatitis [38,39]. A recent large-scale study using the All of Us Research Program reported that 78% of IBD patients had at least one comorbidity, with rheumatologic, dermatologic, and autoimmune conditions being particularly prevalent [40]. The clustering of autoimmune diseases in IBD patients likely reflects shared genetic susceptibility, common immunological pathways, and environmental triggers [41]. These observations reinforce the need for multidisciplinary management approaches that address both intestinal and systemic manifestations of immune dysregulation.

### 4.3. Interpretation of Heterogeneity

The consistently high heterogeneity observed across comorbidities (I^2^ > 90% in 15/18 reviews) reflects three interrelated sources. First, variability in diagnostic methods constitutes the primary driver of certainty downgrading: in NAFLD, liver biopsy detected nearly twice as many cases as non-invasive imaging (59% vs. 30%); bronchiectasis prevalence varied 2.4-fold between high-resolution CT and conventional radiography (12% vs. 5%); and *C. difficile* infection rates differed substantially between PCR and enzyme immunoassay. Second, studied populations differ substantially: population-based registries capture the full disease spectrum, whereas tertiary referral centers overrepresent severe, treatment-refractory disease, which predictably affects the prevalence of severity-associated comorbidities such as osteoporosis, anemia, and frailty. Third, geographic variation reflects both genuine epidemiological differences (e.g., NAFLD prevalence 13% in Asia vs. 43% in North America; hepatitis B/C mirroring endemic patterns) and disparities in diagnostic access across healthcare systems.

Notably, extreme heterogeneity constitutes the norm rather than the exception in global prevalence meta-analyses. A recent umbrella review evaluating 53 worldwide prevalence meta-analyses found that 98.1% reported I^2^ ≥ 75% and 88.7% exceeded I^2^ ≥ 90%, concluding that prevalence-specific frameworks should recognize high heterogeneity as an expected characteristic that requires comprehensive exploration rather than elimination [42]. This evidence supports the interpretation that much of the observed variability in our review reflects genuine epidemiological and methodological differences rather than analytical failure. The critical issue is that most included reviews failed to adequately explore heterogeneity sources through prespecified subgroup analyses and meta-regression. Consequently, estimates with very low certainty should not be extrapolated to specific contexts without recognizing that true values may differ substantially from pooled estimates, whereas estimates with moderate certainty (PSC, uveitis, viral hepatitis) allow more reliable application when appropriately stratified.

### 4.4. Implications for Clinical Practice

This umbrella review demonstrates that comorbidities in IBD are frequent across multiple organ systems, with prevalences ranging from 1.8% to 50.6%. For clinicians, this finding underscores the need for a comprehensive patient assessment that extends beyond intestinal disease. However, the predominantly low or very low certainty of evidence—driven by extreme methodological heterogeneity, non-standardized diagnostic criteria, and inconsistent study populations—limits our ability to establish definitive, evidence-based screening protocols for most comorbidities.

The practical implication is that while comorbidity surveillance remains clinically important, the approach must be individualized rather than protocol-driven for most conditions. For example, although anemia has a reported prevalence of 24%, a complete blood count is already routinely incorporated into IBD monitoring for inflammatory-activity assessment and drug-toxicity surveillance. Similarly, while sexual dysfunction shows the highest reported prevalence (50.6%), the very low certainty (I^2^ = 96.3%, heterogeneous instruments) prevents definitive screening recommendations—yet this does not diminish its clinical relevance. Opportunistic assessment through directed questions during follow-up consultations (“How has your quality of life been?” or “Any concerns about sexual function?”) constitutes appropriate clinical practice regardless of epidemiological limitations.

For certain comorbidities, existing clinical guidelines already provide clear recommendations that align with our findings. Baseline hepatic biochemistry, as recommended by ECCO-ESGAR 2019 guidelines [43], allows for the identification of PSC, particularly in patients with ulcerative colitis. Serological screening for hepatitis B and C before initiating immunosuppressants is a mandatory standard practice [44]. Directed anamnesis for ocular symptoms with ophthalmological referral when indicated follows ECCO 2024 guidelines for uveitis detection [4]. Bone densitometry in patients with specific risk factors (age >65 years, corticosteroid use >3 months, body mass index <20 kg/m^2^) reflects current osteoporosis management guidelines [44]. These established practices do not require modification based on our findings.

For comorbidities with very low certainty, clinical vigilance without systematic screening represents a pragmatic approach. While some reviews report high prevalences (NAFLD 32%, spondyloarthritis 10–13%, bronchiectasis 5–12%), the extreme heterogeneity in diagnostic methods prevents reliable estimates. For instance, NAFLD prevalence varied from 30% with imaging to 59% with biopsy; bronchiectasis from 5% with radiography to 12% with high-resolution CT. Until studies with standardized protocols validate these estimates, targeted evaluation based on clinical suspicion and individual risk factors is more appropriate than universal screening.

The transition toward comprehensive IBD care should be evidence-informed and resource-conscious. We recommend individualized comorbidity assessment based on patient risk profiles, clinical context, and existing guideline recommendations, rather than applying screening protocols not yet supported by sufficient evidence. This approach acknowledges the real burden of comorbidities in IBD while recognizing current methodological limitations and optimizing healthcare resources. Future research employing harmonized diagnostic criteria and robust statistical methods will be essential to establish evidence-based screening protocols.

### 4.5. Strengths and Limitations

This umbrella review has several methodological strengths. First, we conducted a comprehensive search across four major databases without language restrictions to minimize the risk of missing relevant systematic reviews. Second, we employed dual quality assessment using both AMSTAR-2 and ROBIS, providing complementary perspectives on methodological confidence and risk of bias. Third, we applied an adapted GRADE framework specifically designed for prevalence studies, systematically evaluating certainty across five domains and providing transparent justification for each downgrade. Fourth, we implemented predefined hierarchical criteria to select a single systematic review per comorbidity, thereby avoiding the combination of potentially overlapping estimates and the associated unit-of-analysis errors. Fifth, we adhered to established reporting guidelines (PRIOR statement) and methodological frameworks (JBI) for umbrella reviews, enhancing transparency and reproducibility. Finally, the broad scope encompassing 18 comorbidities across multiple organ systems provides clinicians with a consolidated resource for understanding the systemic burden of IBD.

This umbrella review presents several limitations affecting the interpretation of findings. First, although this umbrella review lacks prospective registration, the actions described in the methodology section were adopted to promote transparency, enable critical analysis, and facilitate replication of results. Second, extreme heterogeneity (I^2^ > 80–90%) in most meta-analyses limits the validity of pooled estimates and their transportability to specific populations. Third, most included reviews presented critical methodological deficiencies in AMSTAR-2 (absence of a priori protocols, inconsistent publication bias assessment), which reduced confidence in the estimates.

Fourth, overlap of primary studies was not formally quantified using indices such as CCA, although one review per comorbidity was prioritized to minimize double-counting. Fifth, restriction to English and Spanish may have excluded relevant evidence published in other languages, particularly from Asia, where IBD epidemiology is changing rapidly. Sixth, inclusion was limited to adult populations, excluding relevant pediatric evidence. This is particularly important for comorbidities such as osteoporosis and nutritional deficiencies, where the impact of early IBD diagnosis on growth and bone development is critical.

Fifth, although we applied an adapted GRADE framework to assess the certainty of prevalence estimates, this methodology remains under active development, and its complete standardization has not been achieved, particularly for observational frequency studies. Different groups have proposed slightly different approaches [12,14], and there is no universal consensus on specific thresholds for downgrades in each domain. Finally, important confounders such as disease duration, treatment exposure (particularly corticosteroids and immunosuppressants), and healthcare access were inconsistently reported or adjusted for across included systematic reviews, which may influence prevalence estimates and limit comparability between studies.

## 5. Conclusions

This umbrella review demonstrates that comorbidities in IBD are frequent and clinically significant, supporting systematic comorbidity assessment in patients with IBD. The certainty of evidence is predominantly low or very low due to extreme methodological heterogeneity, non-standardized diagnostic criteria, and limited statistical robustness across included reviews. However, these methodological limitations do not preclude the clinical relevance of comorbidity screening; rather, they highlight the need for improved research quality.

For clinical practice, we recommend individualized comorbidity surveillance based on patient risk profiles and clinical context. For future research, we strongly recommend employing harmonized diagnostic protocols, standardized statistical methods including prespecified subgroup and sensitivity analyses, prospective multicenter cohorts with adequate sample sizes, and validation across diverse populations. These methodological improvements are essential to upgrade evidence certainty and establish robust, evidence-based screening protocols that optimize care for IBD patients.

## Figures and Tables

**Table 1:** Characteristics of Included Systematic Reviews.

Author (Year)	Comorbidity	No. Primary Studies	Sample Size	Regions	Population	Design	IBD Assessment	Comorbidity Assessment	Key Results	Heterogeneity (I^2^)
Lin et al. (2021) [16]	Non-alcoholic fatty liver disease (NAFLD)	27 (13 imaging; 8 elastography; 3 biopsy)	7649	North America, Europe, Asia	Adults with IBD (UC, CD)	SR + MA	Clinical and histological diagnosis of IBD in primary studies	NAFLD * by imaging, transient elastography, biopsy, or serum indices	Pooled NAFLD prevalence: 32% (24–40%). By method: imaging 30% (22–37%), elastography 33% (26–40%), biopsy 59% (20–98%). Advanced fibrosis: 10.3% (5.6–15).	Overall 98%; imaging 99%; elastography 82%; biopsy 96%
Barberio et al. (2021) [15]	Primary sclerosing cholangitis (PSC)	64	776,700	30 countries: the Americas, Europe, the Middle East, Asia, and Africa	Adults ≥ 16 years with IBD (UC, CD, IBD-U)	SR + MA	Clinical and histological/radiological confirmation of IBD	PSC by biochemistry + MRCP/ERCP ± biopsy; or clinical codes	Overall PSC prevalence in IBD: 2.16% (1.76–2.60). By type: UC 2.47% (1.92–3.08), CD 0.96% (0.69–1.28), IBD-U 5.01% (1.26–11.08).	Overall 99.1%; UC 99.2%; CD 91.7%
Shah et al. (2019) [28]	Small intestinal bacterial overgrowth (SIBO)	11	1175	Italy, India, Canada, Belgium, South Korea, Brazil, Spain, Germany	Adults with IBD (UC, CD)	SR + MA	Clinical/histological diagnosis of IBD	SIBO by breath test (glucose, lactulose, 14C-glycocholate)	Overall SIBO prevalence: 22.3% (19.9–24.7). CD: 25.4% (22.5–28.3); UC: 14.3% (10.5–18.1).	Overall 34%; CD 59%; UC 16%
Amakye et al. (2025) [17]	Clostridioides difficile infection (CDI)	28	796,244	North America, Europe, Asia	Adults with IBD	SR + MA	Clinical-endoscopic criteria for IBD	CDI by EIA, PCR, culture, or combinations	Overall CDI prevalence: 8.84% (5.91–13.03). Asia 11.31% (6.70–18.44), North America 7.85% (3.80–15.51), Europe 7.92% (3.87–15.51).	Overall 99%
Giri et al. (2023) [23]	Hepatitis B and C (markers)	34	17,022 (HBsAg), 12,265 (anti-HBc), 10,304 (anti-HCV), 7447 (HCV-RNA)	Asia, Europe, the Americas, and Africa	Adults with IBD	SR + MA	Clinical diagnosis of IBD	Serologies/DNA/RNA for HBV/HCV	HBsAg 3.3% (2.5–4.0); Anti-HBc 14.2% (10.6–17.8); Anti-HCV 1.8% (1.2–2.4); HCV-RNA 0.8% (0.4–1.3).	High (≥75% in several outcomes)
Lin et al. (2024) [19]	Uveitis	21 (uveitis); 14 (episcleritis); 2 (conjunctivitis)	190,941	Europe, Asia, Latin America	Adults with IBD	SR + MA	Clinical diagnosis of IBD	Ophthalmological confirmation or clinical records	Pooled uveitis prevalence: 2.38% (1.60–3.17) in IBD. CD 3.27%; UC 1.60%. Geographic differences (lower in Asia than Europe).	>80%
Karreman et al. (2017) [20]	Axial and peripheral spondyloarthritis	71 (multiple estimates)	NR	Europe, Asia, Americas, Africa	Adults with IBD	SR + MA	Medical history/clinical criteria	Modified NY criteria, ASAS 2009/2011; imaging (X-ray, MRI, US)	Sacroiliitis IBD 10% (8–12); Ankylosing spondylitis 3% (2–4); Peripheral arthritis 13% (12–15).	High (81–94%)
Filmann et al. (2014) [21]	Anemia (iron deficiency and chronic disease)	6	2192	Europe	Adults with IBD	SR + MA	CDAI, HBI, CAI, SCCAI indices	WHO criteria Hb < 13 g/dL (M)/<12 g/dL (F); severe anemia Hb < 10 g/dL; ferritin and transferrin saturation	Total IBD anemia 24% (18–31); CD 27% (19–35); UC 21% (15–27); Severe anemia 2.75% (1.4–4.5).	Overall 91%; CD 90%; UC 80%; severe 80%
Kamali Dolatabadi et al. (2021) [22]	AIEC (adhesion/invasion)	13	465	Europe, Americas, Asia, Oceania	Adults with IBD	SR + MA	Confirmed medical diagnosis	Adhesion/invasion assays in epithelium; macrophages	AIEC prevalence: IBD 28% (18–39); CD 29% (20–40); UC 13% (1–30).	NR
Ma et al. (2024) [26]	Bronchiectasis	16 (sensitivity HRCT: 8)	92,191 (according to sensitivity criteria)	USA, Japan, Greece, Canada, France, Turkey, UK, India, Poland	Adults with IBD	SR + MA	NR	X-ray, CT, HRCT	Overall prevalence: 5% (2–12). Sensitivities: HRCT 12% (4–39); excluding radiography: 9% (3–27).	Overall 100% (high)
Olfati et al. (2023) [30]	Migraine	10	62,554	Europe, Asia, the Middle East, and North America	Adults with IBD	SR + MA (cross-sectional)	Clinical diagnosis of IBD	Migraine diagnosis according to each study; questionnaires/registry	Overall prevalence: 19% (15–22). UC 10% (4–15); CD 24% (17–30). OR IBD vs. controls 1.51 (1–2.27).	Very high (I^2^ ≈ 99%)
Shi et al. (2025) [31]	Fecal incontinence	15 (prevalence)	7232 (for prevalence)	Global (United States, Europe, Australia, Asia)	IBD adults and pediatrics (some studies)	SR + MA	IBD diagnosis according to each study	Clinical definitions or incontinence questionnaires; sometimes FIQL/CCF-FI	Overall FI prevalence in CD: 34.8% (CI 27.9–41.9), with very high heterogeneity; several significant predictors (e.g., previous surgery).	Overall ~ 99%
Nardone et al. (2025) [32]	Sexual dysfunction (overall, FSD and ED)	18	2694	13 countries (single-country studies)	Adults with IBD	SR + MA	Histology or imaging for IBD	Validated questionnaires (FSFI, IIEF, ASEX, BISF-W)	Overall SD prevalence in IBD: 50.6% (40.8–60.5; I^2^ 96.3). UC 64.8% (45.1–82.1); CD 58.3% (36.0–79.0). Women 62.7% vs. men 34.0%. Active 75.1% vs. inactive 34.2%.	Very high (88–96% by subgroups)
Shen et al. (2024) [27]	Metabolic syndrome	11 (9 for overall pooling)	2501	United States, Serbia, Italy, Portugal, Japan, South Korea, Turkey	Adults with IBD (30–52 years)	SR + MA	ECCO guidelines/clinical criteria	ATP-III, IDF, AHA/NHLBI	Combined MS prevalence: 19.4% (15.1–23.8). UC 38.2% (20.4–59.9); CD 13.6% (6.4–26.7). Sensitivity: 21.9% (18–25.8).	Overall 81% (decreases in sensitivity)
Marzban Abbas Abadi et al. (2025) [29]	Osteoporosis	24	417,298	21 countries: Algeria, Australia, China (n = 2), Denmark, Germany, Iran, Japan, South Korea, Mexico, The Netherlands, Poland (n = 2), Portugal, Romania, Spain (n = 2), Taiwan, Tunisia (n = 2), Turkey, United States (n = 2)	Adults (≥18 years) with IBD (Crohn’s, ulcerative colitis); criteria according to each study; n ≥ 100 per study	Systematic review and meta-analysis of prevalence (PRISMA)	Diagnosis according to each study (ICD codes/registries/clinical criteria) and by type (CD, UC)	Osteoporosis by DEXA (predominant), ICD codes, and CT	Overall osteoporosis prevalence in IBD: 12.2% (95% CI 9.1–15.3); OR vs. controls: 1.64 (95% CI 1.24–2.16); CD 14.9% (8.8–20.9) vs. UC 11.4% (5.8–17.0) (no difference); Men 9.6% (3.0–16.3); Women 10.5% (6.8–14.1).	Overall 99.7%; OR 70.9%; by subgroups see ‘Prevalences’ sheet
Huang et al. (2022) [24]	Frailty	9	1,495,695	Mainly USA and Sweden	Adults with IBD	SR + MA	Clinical codes/registry (ICD 9/10) and clinical diagnoses according to each study	Validated frailty indices: HFRS, CFI, SFI, mFI; scales based on claims and clinical records	Frailty prevalence: 18% (95% CI 12–24); associated with all-cause mortality (HR ≈ 2.25) and major adverse events (adjusted estimates), details in supplementary tables	99.9% for pooled prevalence
Kiliç et al. (2024) [25]	“≥1” EIM (articular—axial and peripheral—, ocular, and cutaneous)	52	352,454	Europe, Asia, North America, Middle East	Adults with IBD	SR + MA	UC/CD according to each study (cohort and population-based)	Clinical/imaging/registry definitions for EIM; duplicate extraction; JBI quality threshold ≥ 7/9	“≥1” EIM: 24% in IBD (95% CI 19–31), 27% in UC, 35% in CD; axial 4–5%, peripheral 11–17%, ocular 2–3%, erythema nodosum 1–3%, and pyoderma gangrenosum ~ 1% (by phenotype)	Overall 100%; UC 98%; CD 97%; axial/peripheral/ocular and cutaneous 97–99%
Alinaghi et al. (2020) [18]	Psoriasis and psoriatic arthritis	97 (for prevalence, 67)	199,672 (psoriasis prevalence); subsamples by phenotype/age	Europe, Asia, North America	Adults and pediatrics with IBD (UC/CD)	SR + MA	Clinical/histological/registry diagnosis according to each study	Psoriasis by clinical or dermatological diagnosis; PsA by clinical/rheumatological criteria	Psoriasis in IBD: 4.2% (95% CI 3.4–5.0). In CD: 3.6%; in UC: 2.8%. Adults 3.8% vs. <18 years 8.4%. PsA in IBD ≈ 1.0% (95% CI 0–1.9).	Overall ≈ 98%

Legend/Notes. SR: systematic review; MA: meta-analysis; IBD: inflammatory bowel disease; CD: Crohn’s disease; UC: ulcerative colitis; IBD-U: IBD unclassified; 95% CI: 95% confidence interval; I^2^: heterogeneity (0–25% low; 26–50% moderate; 51–75% high; 76–100% very high); OR: odds ratio; NAFLD: non-alcoholic fatty liver disease; PSC: primary sclerosing cholangitis; SIBO: small intestinal bacterial overgrowth; CDI: Clostridioides difficile infection; AIEC: adherent-invasive *E. coli*; EIM: extraintestinal manifestations; HRCT: high-resolution computed tomography; CT: computed tomography; MRI: magnetic resonance imaging; X-ray: plain radiography; MRCP: magnetic resonance cholangiopancreatography; ERCP: endoscopic retrograde cholangiopancreatography; PCR: polymerase chain reaction (molecular); EIA: enzyme immunoassay; HBsAg: hepatitis B surface antigen; anti-HBc: antibodies against HBV core; anti-HCV: antibodies against HCV; HCV-RNA: detectable HCV ribonucleic acid; DEXA: dual-energy X-ray absorptiometry; Hb: hemoglobin; NR: not reported. Prevalences correspond to pooled estimates (when applicable, random-effects model) or ranges when pooling was not performed; when subgroups by method/continent/phenotype are shown, this is explicitly indicated in “Key results”. * Now termed metabolic dysfunction-associated steatotic liver disease (MASLD) according to 2023 multi-society Delphi consensus; original terminology retained as included studies predate nomenclature change.

**Table 2:** Assessment of Certainty of Evidence (Adapted GRADE) for Prevalence Estimates of Comorbidities in IBD.

Comorbidity	Author (Year)	Prevalence (95% CI)	No. Studies/No. Patients	Risk of Bias ^1^	Inconsistency ^2^	Indirectness ^3^	Imprecision ^4^	Publication Bias ^5^	Final Certainty	Justification for Downgrades
NAFLD	Lin 2021 [16]	32% (24–40)	27/7649	⊖ Serious (-1)	⊖⊖ Very serious (-2)	⊖ Serious (-1)	Not serious	⊖ Serious (-1)	⊝⊝◯◯ VERY LOW	Heterogeneous diagnostic method (imaging 30%, elastography 33%, biopsy 59%); I^2^ = 98%; extreme geographic variation (Asia 13% vs. North America 43%); pre-MASLD 2023 definition; asymmetric funnel; many conference abstracts
PSC	Barberio 2021 [15]	2.16% (1.76–2.60)	64/776,700	⊖ Serious (-1)	⊖ Serious (-1)	Not serious	Not serious	Not assessed	⊝⊝⊝◯ MODERATE	Mixed methods: ICD codes vs. MRCP/ERCP/biopsy; I^2^ = 99.1% overall but <50% in subgroups by rigorous diagnostic method; very large N compensates for imprecision
SIBO	Shah 2019 [28]	22.3% (19.9–24.7)	11/1175	⊖ Serious (-1)	⊖ Serious (-1)	⊖ Serious (-1)	Not serious	Not reported	⊝⊝◯◯ LOW	Heterogeneous breath tests (glucose, lactulose, 14C-glycocholate); I^2^ = 34%; populations with different IBD activity; moderate n
*C. difficile*	Amakye 2025 [17]	8.84% (5.91–13.03)	28/796,244	⊖ Serious (-1)	⊖⊖ Very serious (-2)	Not serious	⊖ Serious (-1)	Not serious	⊝⊝◯◯ LOW	Heterogeneous diagnostic methods (PCR higher sensitivity than EIA); I^2^ = 99%; wide CI (5.91–13.03, spans 7.12 points); very large N but extreme heterogeneity; bias assessed (Egger non-significant)
Hepatitis B (HBsAg+)	Giri 2023 [23]	3.3% (2.5–4.0)	34/17,022	Not serious	⊖ Serious (-1)	Not serious	Not serious	Not serious	⊝⊝⊝◯ MODERATE	Standardized serological methods; I^2^ = 75%; expected geographic variation (endemic in Asia); narrow CI; bias assessed (Egger)
Hepatitis C (anti-HCV)	Giri 2023 [23]	1.8% (1.2–2.4)	34/10,304	Not serious	⊖ Serious (-1)	Not serious	Not serious	Not serious	⊝⊝⊝◯ MODERATE	Standardized serological methods; I^2^ ≥ 75%; consistently low prevalence; acceptable CI
Uveitis	Lin 2024 [19]	2.38% (1.60–3.17)	14/115,854	Not serious	⊖ Serious (-1)	Not serious	Not serious	Not assessed	⊝⊝⊝◯ MODERATE	Ophthalmological confirmation in 8/14 studies; I^2^ > 80% overall but lower in subgroups (CD vs. UC); geographic differences explained (Asia <Europe); large N
Axial spondyloarthritis (sacroiliitis)	Karreman 2017 [20]	10% (8–12)	71 (multiple estimates)	⊖⊖ Very serious (-2)	⊖⊖ Very serious (-2)	⊖ Serious (-1)	Not serious	Not assessed	⊝◯◯◯ VERY LOW	Heterogeneous diagnostic criteria (modified NY, ASAS, clinical alone); I^2^ = 81–94%; variable imaging methods (X-ray, MRI, CT); old studies (2000–2015); “spondyloarthritis” definition not standardized
Spondyloarthritis (peripheral arthritis)	Karreman 2017 [20]	13% (12–15)	71	⊖⊖ Very serious (-2)	⊖⊖ Very serious (-2)	⊖ Serious (-1)	Not serious	Not assessed	⊝◯◯◯ VERY LOW	Non-standardized clinical definitions; I^2^ = 81–94%; mixture of reactive arthritis, oligoarthritis, polyarthritis; ascertainment bias in rheumatology centers
Anemia	Filmann 2014 [21]	24% (18–31)	6/2192	Not serious	⊖⊖ Very serious (-2)	⊖ Serious (-1)	Not serious	Not assessed	⊝⊝◯◯ LOW	Standardized WHO/ECCO definition; I^2^ = 91%; heterogeneity partially explained by IBD activity, but not always adjusted in analysis; old data (2009–2012); Europe only (not generalizable)
AIEC	Kamali Dolatabadi 2021 [22]	28% (18–39)	13/465	⊖⊖ Very serious (-2)	⊖ Serious (-1)	⊖ Serious (-1)	⊖⊖ Very serious (-2)	Not assessed	⊝◯◯◯ VERY LOW	Very heterogeneous in vitro laboratory methods (adhesion/invasion without standardized protocols); I^2^ not reported but very wide ranges; small biopsy samples; n = 465 total (very low); very wide CI (21 points); selection bias (centers with AIEC expertise)
Bronchiectasis	Ma 2024 [26]	5% (2–12) [HRCT: 12% (4–39)]	16/92,191	⊖⊖ Very serious (-2)	⊖⊖ Very serious (-2)	⊖ Serious (-1)	⊖ Serious (-1)	Not serious	⊝◯◯◯ VERY LOW	Critical diagnostic method: X-ray (low sensitivity) vs. HRCT (gold standard); I^2^ = 100%; prevalence varies 6× by method (X-ray 5% vs. HRCT 39%); many studies without systematic HRCT; very wide CIs; bias assessed (Egger)
Migraine	Olfati 2023 [30]	19% (15–22)	10/62,554	⊖⊖ Very serious (-2)	⊖⊖ Very serious (-2)	⊖ Serious (-1)	Not serious	Not assessed	⊝◯◯◯ VERY LOW	Heterogeneous migraine definitions (IHS criteria vs. non-validated questionnaires); all cross-sectional (do not capture incidence); I^2^ ≈ 99%; high recall bias; no adjustment for headache-causing medications (anti-TNF)
Fecal incontinence (CD)	Shi 2025 [31]	34.8% (27.9–41.9)	15/7232	⊖ Serious (-1)	⊖⊖ Very serious (-2)	⊖ Serious (-1)	Not serious	Not assessed	⊝⊝◯◯ LOW	Heterogeneous definitions (clinical vs. FIQL/CCF-FI questionnaires); I^2^ ≈ 99%; CD predominance (UC evidence insufficient); selection bias (post-surgery patients overrepresented)
Sexual dysfunction	Nardone 2025 [32]	50.6% (40.8–60.5)	18/2694	⊖⊖ Very serious (-2)	⊖⊖ Very serious (-2)	⊖ Serious (-1)	⊖ Serious (-1)	Not serious	⊝◯◯◯ VERY LOW	Very heterogeneous questionnaires (FSFI, IIEF, ASEX, BISF-W not directly comparable); I^2^ = 96.3%; small n (2694 total); very wide CI (20 points); selection bias (patients who agree to answer sexual questionnaires); participation bias; bias assessed (Egger)
Metabolic syndrome	Shen 2024 [27]	19.4% (15.1–23.8)	11/2501	⊖ Serious (-1)	⊖⊖ Very serious (-2)	⊖ Serious (-1)	Not serious	Partially assessed	⊝⊝◯◯ LOW	Heterogeneous diagnostic criteria (ATP-III, IDF, AHA/NHLBI); I^2^ = 81% (attenuated to 52% in sensitivity); unexplained UC 38.2% vs. CD 13.6% discrepancy; possible confounding by corticosteroids
Osteoporosis	Marzban Abbas Abadi 2025 [29]	12.2% (9.1–15.3)	24/417,298	⊖ Serious (-1)	⊖⊖ Very serious (-2)	Not serious	Not serious	Not assessed	⊝⊝◯◯ LOW	Mixed DEXA (gold standard) vs. ICD codes (low specificity); I^2^ = 99.7%; meta-regression did not identify significant covariates (age, % corticosteroids); enormous N compensates for imprecision
Frailty	Huang 2022 [24]	18% (12–24)	9/1,495,695	⊖ Serious (-1)	⊖⊖ Very serious (-2)	⊖ Serious (-1)	Not serious	Not serious	⊝⊝◯◯ LOW	Multiple scales (HFRS, CFI, SFI, mFI) not directly comparable; I^2^ = 99.9%; “frailty” definition not standardized in IBD; USA/Sweden predominance (not generalizable); bias assessed (Egger/Begg)
≥1 Extraintestinal manifestation	Kiliç 2024 [25]	24% (19–31) [UC: 27%; CD: 35%]	52/352,454	⊖ Serious (-1)	⊖⊖ Very serious (-2)	Not serious	Not serious	Not serious	⊝⊝◯◯ LOW	Heterogeneous EIM definitions between studies; I^2^ = 100%; JBI quality threshold ≥ 7/9 applied but capture methods vary; overlap between EIMs not always reported; bias assessed (Egger/Begg)
Psoriasis	Alinaghi 2020 [18]	4.2% (3.4–5.0) [Adults: 3.8%; <18 years: 8.4%]	97/199,672	⊖ Serious (-1)	⊖⊖ Very serious (-2)	⊖ Serious (-1)	Not serious	Not serious	⊝⊝◯◯ LOW	Mixed dermatological diagnosis vs. administrative codes; I^2^ ≈ 98%; age differences (pediatric vs. adult) suggest different populations; ascertainment bias (patients with IBD more monitored); bias assessed (Egger)
Psoriatic arthritis	Alinaghi 2020 [18]	1.0% (0–1.9)	97/subsamples	⊖⊖ Very serious (-2)	⊖ Serious (-1)	⊖ Serious (-1)	⊖⊖ Very serious (-2)	Not assessed	⊝◯◯◯ VERY LOW	Heterogeneous rheumatological criteria (CASPAR vs. clinical); I^2^ ≈ 98%; CI includes null (0–1.9%); small n per comorbidity; referral bias (only referred to rheumatology)

Symbols and notation: ⊖ indicates downgrade (certainty reduction); the number in parentheses indicates levels of downgrade applied (-1 = one-level reduction; -2 = two-level reduction). Initial certainty for observational studies: Low (⊝⊝◯◯); after downgrades can decrease to Very Low (⊝◯◯◯). Upgrades were not applied in this study. I^2^: statistical heterogeneity (values > 75% considered very high). Footnotes: ^1^ Risk of bias: Assessment of sampling methods, population representativeness, and validation of diagnostic definitions. ^2^ Inconsistency: Statistical heterogeneity (I^2^), breadth of prevalence ranges, degree of explanation through subgroup analyses. ^3^ Indirectness: Differences between the studied populations and the target population, obsolescence of diagnostic definitions, and representativeness of healthcare settings. ^4^ Imprecision: Width of 95% confidence intervals relative to point estimate and total sample size. ^5^ Publication bias: Formal assessment through statistical tests (funnel plot, Egger/Begg test) when performed in included systematic reviews.

## Data Availability

All relevant data are within the manuscript and its Supplementary Materials.

## Supplementary Materials

The following supporting information can be downloaded at: https://www.mdpi.com/article/10.3390/jcm15051739/s1, Table S1: PRISMA 2020 Main Checklist; Table S2: Search strategy; Table S3: Systematic Reviews Excluded for Sharing Objectives with Included Studies; Table S4: Methodological Quality Assessment (AMSTAR-2); Table S5: Risk of Bias Assessment Using ROBIS; Figure S1: Flowchart of Study Selection. Reference [45] are cited in the supplementary materials.
