# Supplementary material for: Prevalence of Comorbidities in Inflammatory Bowel Disease: An Umbrella Review of 18 Systematic Reviews
Source: J Clin Med. 2026 Feb 25;15(5):1739. doi: 10.3390/jcm15051739 (PMC12986176; doi:10.3390/jcm15051739)
Supplement: Supplementary file 1 [file jcm-15-01739-s001.zip › jcm-4105088-supplementary.pdf]

Table S1. PRISMA 2020 Main Checklist

Topic	No.	Item	Location where item is reported
**TITLE**			
**Title**	1	Identify the report as a systematic review.	1
**ABSTRACT**			
**Abstract**	2	See the PRISMA 2020 for Abstracts checklist	
**INTRODUCTION**			
**Rationale**	3	Describe the rationale for the review in the context of existing knowledge.	2
**Objectives**	4	Provide an explicit statement of the objective(s) or question(s) the review addresses.	3
**METHODS**			
**Eligibility criteria**	5	Specify the inclusion and exclusion criteria for the review and how studies were grouped for the syntheses.	4
**Information sources**	6	Specify all databases, registers, websites, organisations, reference lists and other sources searched or consulted to identify studies. Specify the date when each source was last searched or consulted.	5
**Search strategy**	7	Present the full search strategies for all databases, registers and websites, including any filters and limits used.	5-6
**Selection process**	8	Specify the methods used to decide whether a study met the inclusion criteria of the review, including how many reviewers screened each record and each report retrieved, whether they worked independently, and if applicable, details of automation tools used in the process.	6-7
**Data collection process**	9	Specify the methods used to collect data from reports, including how many reviewers collected data from each report, whether they worked independently, any processes for obtaining or confirming data from study investigators, and if applicable, details of automation tools used in the process.	6-7
**Data items**	10a	List and define all outcomes for which data were sought. Specify whether all results that were compatible with each outcome domain in each study were sought (e.g. for all measures, time points, analyses), and if not, the methods used to decide which results to collect.	6-7
	10b	List and define all other variables for which data were sought (e.g. participant and intervention characteristics, funding sources). Describe any assumptions made about any missing or unclear information.	7
**Study risk of bias assessment**	11	Specify the methods used to assess risk of bias in the included studies, including details of the tool(s) used, how many reviewers assessed each study and whether they worked independently, and if applicable, details of automation tools used in the process.	7
**Effect measures**	12	Specify for each outcome the effect measure(s) (e.g. risk ratio, mean difference) used in the synthesis or presentation of results.	7

Topic	No.	Item	Location where item is reported
**Synthesis methods**	13a	Describe the processes used to decide which studies were eligible for each synthesis (e.g. tabulating the study intervention characteristics and comparing against the planned groups for each synthesis (item 5)).	7
	13b	Describe any methods required to prepare the data for presentation or synthesis, such as handling of missing summary statistics, or data conversions.	7
	13c	Describe any methods used to tabulate or visually display results of individual studies and syntheses.	7
	13d	Describe any methods used to synthesize results and provide a rationale for the choice(s). If meta-analysis was performed, describe the model(s), method(s) to identify the presence and extent of statistical heterogeneity, and software package(s) used.	7
	13e	Describe any methods used to explore possible causes of heterogeneity among study results (e.g. subgroup analysis, meta-regression).	7
	13f	Describe any sensitivity analyses conducted to assess robustness of the synthesized results.	-
**Reporting bias assessment**	14	Describe any methods used to assess risk of bias due to missing results in a synthesis (arising from reporting biases).	8
**Certainty assessment**	15	Describe any methods used to assess certainty (or confidence) in the body of evidence for an outcome.	8
**RESULTS**			
**Study selection**	16a	Describe the results of the search and selection process, from the number of records identified in the search to the number of studies included in the review, ideally using a flow diagram.	8
	16b	Cite studies that might appear to meet the inclusion criteria, but which were excluded, and explain why they were excluded.	8
**Study characteristics**	17	Cite each included study and present its characteristics.	9
**Risk of bias in studies**	18	Present assessments of risk of bias for each included study.	10
**Results of individual studies**	19	For all outcomes, present, for each study: (a) summary statistics for each group (where appropriate) and (b) an effect estimate and its precision (e.g. confidence/credible interval), ideally using structured tables or plots.	11
**Results of syntheses**	20a	For each synthesis, briefly summarise the characteristics and risk of bias among contributing studies.	12
	20b	Present results of all statistical syntheses conducted. If meta-analysis was done, present for each the summary estimate and its precision (e.g. confidence/credible interval) and measures of statistical heterogeneity. If comparing groups, describe the direction of the effect.	12-18
	20c	Present results of all investigations of possible causes of heterogeneity among study results.	12-18
	20d	Present results of all sensitivity analyses conducted to assess the robustness of the synthesized results.	12-18

Topic	No.	Item	Location where item is reported
**Reporting biases**	21	Present assessments of risk of bias due to missing results (arising from reporting biases) for each synthesis assessed.	12-18
**Certainty of evidence**	22	Present assessments of certainty (or confidence) in the body of evidence for each outcome assessed.	12-18
**DISCUSSION**			
**Discussion**	23a	Provide a general interpretation of the results in the context of other evidence.	18
	23b	Discuss any limitations of the evidence included in the review.	18-24
	23c	Discuss any limitations of the review processes used.	18-24
	23d	Discuss implications of the results for practice, policy, and future research.	18-24
**OTHER INFORMATION**			
**Registration and protocol**	24a	Provide registration information for the review, including register name and registration number, or state that the review was not registered.	5
	24b	Indicate where the review protocol can be accessed, or state that a protocol was not prepared.	5
	24c	Describe and explain any amendments to information provided at registration or in the protocol.	5
**Support**	25	Describe sources of financial or non-financial support for the review, and the role of the funders or sponsors in the review.	8
**Competing interests**	26	Declare any competing interests of review authors.	8
**Availability of data, code and other materials**	27	Report which of the following are publicly available and where they can be found: template data collection forms; data extracted from included studies; data used for all analyses; analytic code; any other materials used in the review.	8

PRIMSA Abstract Checklist

Topic	No.	Item	Reported?
**TITLE**			
**Title**	1	Identify the report as a systematic review.	Yes
**BACKGROUND**			
**Objectives**	2	Provide an explicit statement of the main objective(s) or question(s) the review addresses.	Yes
**METHODS**			

Topic	No.	Item	Reported?
**Eligibility criteria**	3	Specify the inclusion and exclusion criteria for the review.	Yes
**Information sources**	4	Specify the information sources (e.g. databases, registers) used to identify studies and the date when each was last searched.	Yes
**Risk of bias**	5	Specify the methods used to assess risk of bias in the included studies.	Yes
**Synthesis of results**	6	Specify the methods used to present and synthesize results.	Yes
**RESULTS**			
**Included studies**	7	Give the total number of included studies and participants and summarise relevant characteristics of studies.	Yes
**Synthesis of results**	8	Present results for main outcomes, preferably indicating the number of included studies and participants for each. If meta-analysis was done, report the summary estimate and confidence/credible interval. If comparing groups, indicate the direction of the effect (i.e. which group is favoured).	Yes
**DISCUSSION**			
**Limitations of evidence**	9	Provide a brief summary of the limitations of the evidence included in the review (e.g. study risk of bias, inconsistency and imprecision).	Yes
**Interpretation**	10	Provide a general interpretation of the results and important implications.	Yes
**OTHER**			
**Funding**	11	Specify the primary source of funding for the review.	Yes
**Registration**	12	Provide the register name and registration number.	Yes

From: Page MJ, McKenzie JE, Bossuyt PM, Boutron I, Hoffmann TC, Mulrow CD, et al. The PRISMA 2020 statement: an updated guideline for reporting systematic reviews. MetaArXiv. 2020, September 14. DOI: 10.31222/osf.io/v7gm2. For more information, visit: www.prisma-statement.org

PRIOR statement—a reporting guideline for overviews of reviews

Section	Item No.	PRIOR Item	Compliant	Location (page/section)
TITLE	1	Identify the report as an overview of reviews.	Yes	Title: 'Umbrella review of systematic reviews'
ABSTRACT	2	Provide an accurate summary of objectives, methods, and main results.	Yes	Abstract
INTRODUCTION	3	Explain rationale in context of existing knowledge.	Yes	Introduction (first 4 paragraphs)

INTRODUCTION	4	Clearly state the objectives or questions addressed.	Yes	End of Introduction
METHODS	5a	Specify inclusion and exclusion criteria.	Yes	Selection criteria
METHODS	5b	Define 'systematic review' as used for inclusion.	Partial	Implicit definition: RS with reproducible search and criteria
METHODS	6	Specify databases and date of last search.	Yes	Search strategy – PubMed, Embase, Scopus, WoS (Sept 5, 2025)
METHODS	7	Present full reproducible search strategies.	Yes	Supplementary Material 2
METHODS	8a	Describe selection process for eligible reviews.	Yes	Selection process paragraph
METHODS	8b	Describe identification and management of overlap.	Partial	One review per comorbidity; no formal CCA index
METHODS	9a	Describe data-collection methods.	Yes	Data extraction section
METHODS	9b	Describe management of overlap at primary-study level.	Partial	Addressed qualitatively; no quantitative metric
METHODS	9c	Describe how discrepant data were managed.	Yes	Consensus or third reviewer adjudication
METHODS	10	List and define all variables/outcomes extracted; note assumptions.	Yes	Data extraction section
METHODS	11a	Describe methods to assess risk of bias of included reviews.	Yes	AMSTAR-2 and ROBIS described
METHODS	11b	Describe how RoB of primary studies was collected from reviews.	Yes	Extracted NOS/AHRQ from each review

METHODS	11c	Describe methods to assess RoB of supplemental primary studies.	N/A	Not applicable
METHODS	12a	Describe synthesis methods and rationale.	Yes	Synthesis and selection of main estimate
METHODS	12b	Describe methods to explore heterogeneity.	Yes	Subgroup analyses by diagnostic method, geography, I^2
METHODS	12c	Describe sensitivity analyses.	Yes	Sensitivity analyses within reviews discussed
METHODS	13	Describe assessment of reporting bias.	Yes	Publication bias via funnel/Egger/Begg
METHODS	14	Describe certainty/confidence assessment.	Yes	Certainty of evidence (GRADE adapted for prevalence)
RESULTS	15a	Describe results of search/selection, ideally with flow diagram.	Yes	Study selection + Supplementary Material 3
RESULTS	15b	Provide list of excluded studies with reasons.	Yes	Supplementary Material 4
RESULTS	16	Cite each included review and present its characteristics.	Yes	Table 1
RESULTS	17	Describe extent of primary-study overlap.	Partial	Addressed narratively; no quantitative CCA
RESULTS	18a	Present RoB/quality for each included review.	Yes	AMSTAR-2 (Suppl. 4) and ROBIS (Suppl. 5)
RESULTS	18b	Present RoB of primary studies (collected from reviews).	Yes	Extracted NOS/AHRQ data summarized
RESULTS	18c	Present RoB of supplemental primary studies (if included).	N/A	Not applicable

RESULTS	19a	Summarize evidence for all outcomes with precision/heterogeneity.	Yes	Main prevalences + Table 1 + GRADE Table 2
RESULTS	19b	Report heterogeneity exploration.	Yes	Interpretation of heterogeneity (Discussion)
RESULTS	19c	Present sensitivity analyses.	Yes	Sensitivity results (e.g., Shen 2024 reduced I²)
RESULTS	20	Present assessments of reporting bias for each synthesis.	Yes	AMSTAR-2 section + Supplementary 4
RESULTS	21	Present certainty/confidence in the body of evidence.	Yes	Table 2 (GRADE adapted)
DISCUSSION	22a	Summarize main findings including discrepancies.	Yes	Discussion – Main findings
DISCUSSION	22b	Interpret results in context of other evidence.	Yes	Discussion – Comparison with literature
DISCUSSION	22c	Discuss limitations of evidence and overview methods.	Yes	Limitations section
DISCUSSION	22d	Discuss implications for practice, policy, and future research.	Yes	Implications for practice + Future directions
OTHER INFO	23a	Provide registration details or state unregistered.	No	No prospective registration
OTHER INFO	23b	Indicate where protocol can be accessed or note absence.	Yes	Followed PRIOR and JBI; no protocol registered
OTHER INFO	23c	Describe amendments to registration/protocol.	N/A	Not applicable

OTHER INFO	24	Describe funding sources and sponsor role.	Yes	The publication fee (APC) was covered by Universidad Señor de Sipán (USS).
OTHER INFO	25	Declare competing interests.	Yes	Conflict of interest – none declared
OTHER INFO	26a	Provide corresponding author contact.	Yes	Title page (ORCID + email)
OTHER INFO	26b	Describe author contributions and guarantor.	Yes	Authors' contribution
OTHER INFO	27	Report availability of data, forms, code, materials.	Partial	Data available upon request – no repository

Table S2. Search strategy

Search strategy in PUBMED	
#1	"Inflammatory Bowel Diseases"[Mesh] OR "Crohn Disease"[Mesh] OR "Colitis, Ulcerative"[Mesh] OR "inflammatory bowel disease*"[Title/Abstract] OR "IBD"[Title/Abstract] OR "Crohn*"[Title/Abstract] OR "ulcerative colitis"[Title/Abstract] OR "Crohn disease"[Title/Abstract] OR "Crohn's disease"[Title/Abstract]
#2	"Prevalence"[Mesh] OR "Comorbidity"[Mesh] OR prevalence[Title/Abstract] OR proportion*[Title/Abstract] OR frequency[Title/Abstract] OR rate*[Title/Abstract] OR incidence[Title/Abstract] OR comorbid*[Title/Abstract] OR "co-morbid*"[Title/Abstract]
#3	Filters: "Systematic Review", "Meta-Analysis"
#4	#1 AND #2 AND #3
Search strategy in SCOPUS	
#1	TITLE-ABS-KEY("inflammatory bowel disease*" OR "IBD" OR "Crohn*" OR "ulcerative colitis" OR "Crohn disease" OR "Crohn's disease") AND TITLE-ABS-KEY(prevalence OR proportion* OR frequency OR rate* OR incidence OR comorbid* OR "co-morbid*")
#2	TITLE-ABS-KEY(prevalence OR proportion* OR frequency OR rate* OR incidence OR comorbid* OR "co-morbid*")
#3	TITLE-ABS-KEY ("systematic review" OR "systematic reviews" OR "meta-analysis" OR "meta analysis" OR "metaanalysis" OR "umbrella review" OR "overview of reviews" OR "review of reviews" OR "scoping review" OR "narrative review" OR "pooled analysis")
#4	#1 AND #2 AND #3
Search strategy in Web of Science	
#1	TS=("inflammatory bowel disease*" OR "IBD" OR "Crohn*" OR "ulcerative colitis" OR "Crohn disease" OR "Crohn's disease")
#2	TS=(prevalence OR proportion* OR frequency OR rate* OR incidence OR comorbid* OR "co-morbid*")
#3	TS=("systematic review" OR "systematic reviews" OR "meta-analysis" OR "meta analysis" OR "umbrella review" OR "overview of reviews" OR "review of reviews" OR "scoping review" OR "narrative review")
#4	#1 AND #2 AND #3
Search strategy in EMBASE	
#1	'inflammatory bowel disease'/exp OR 'inflammatory bowel disease':ti,ab,kw OR 'inflammatory bowel diseases':ti,ab,kw OR 'crohn disease'/exp OR 'crohn disease':ti,ab,kw OR 'crohn`s disease':ti,ab,kw OR 'crohns disease':ti,ab,kw OR 'cleron disease':ti,ab,kw OR 'enteritis regionalis':ti,ab,kw OR 'intestinal tract, regional enteritis':ti,ab,kw OR 'morbus crohn':ti,ab,kw OR 'regional enteritis':ti,ab,kw OR 'regional enterocolitis':ti,ab,kw OR 'ulcerative colitis'/exp OR 'chronic ulcerative colitis':ti,ab,kw OR 'colitis ulcerativa':ti,ab,kw OR 'colitis ulcerosa':ti,ab,kw OR 'colitis ulcerosa chronica':ti,ab,kw OR 'colitis, mucosal':ti,ab,kw OR 'colitis, ulcerative':ti,ab,kw OR 'colitis, ulcerous':ti,ab,kw OR 'colon, chronic ulceration':ti,ab,kw OR 'histiocytic ulcerative colitis':ti,ab,kw OR 'mucosal colitis':ti,ab,kw OR 'ulcerative colitis':ti,ab,kw OR 'ulcerative colorectitis':ti,ab,kw OR 'ulcerative procto colitis':ti,ab,kw OR 'ulcerative proctocolitis':ti,ab,kw OR 'ulcerous colitis':ti,ab,kw
#2	'prevalence'/exp OR 'prevalence' OR 'prevalence study'
#3	'systematic review'/exp OR 'review, systematic' OR 'systematic review' OR 'meta analysis'/exp OR 'analysis, meta' OR 'meta analysis' OR 'meta-analysis' OR 'metaanalysis'
#4	#1 AND #2 AND #3

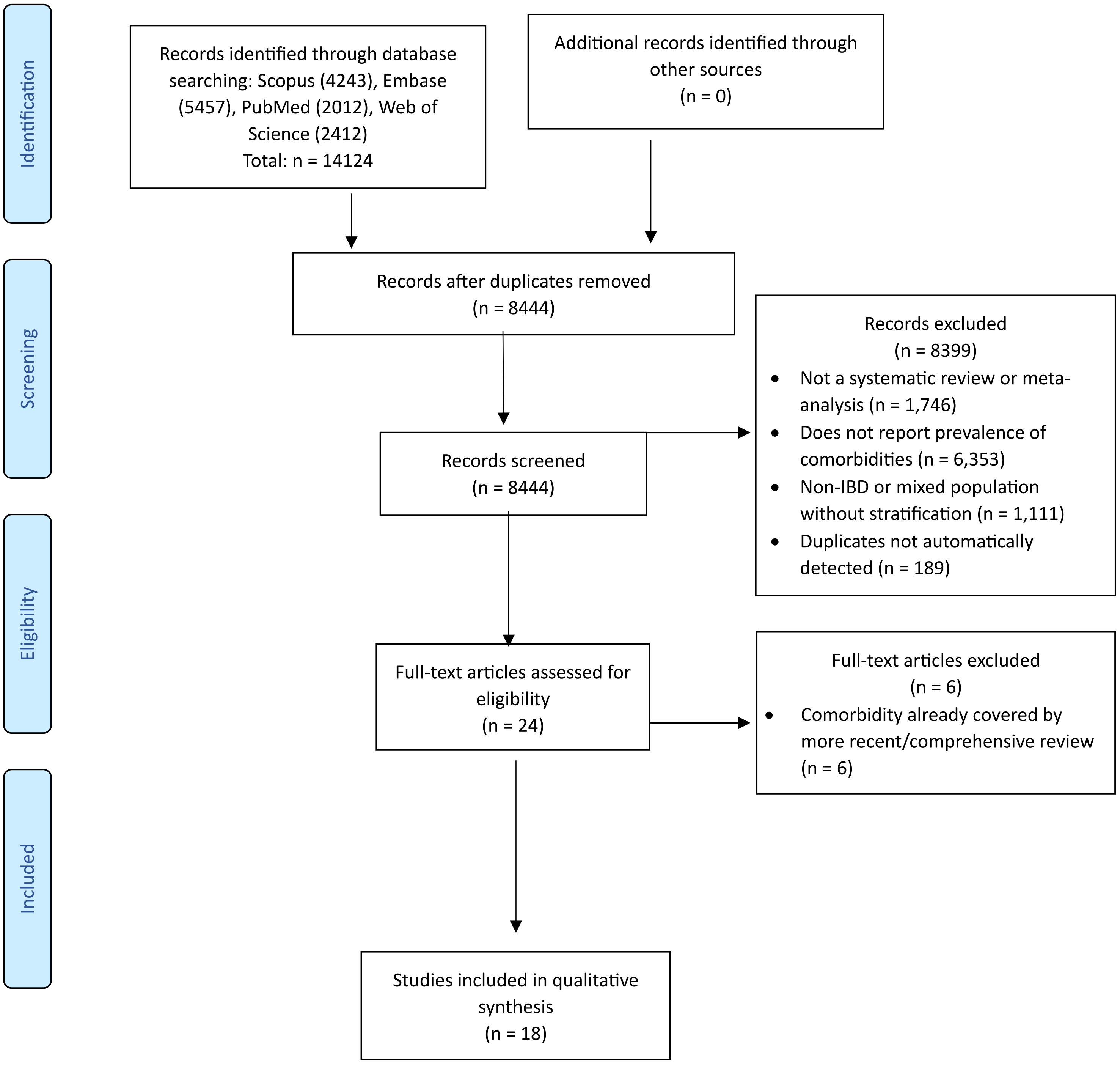

Fig S1. Flowchart of Study Selection

Table S3. Systematic Reviews Excluded for Sharing Objectives with Included Studies

**Comorbidity**	**Excluded study (author, year)**	**Title of excluded study**	**No. studies (excluded)**	**No. patients (excluded)**	**Included study (author, year)**	**Title of included study**	**No. studies (included)**	**No. patients (included)**	**Main justification**	**Criteria applied**
NAFLD (non-alcoholic fatty liver disease)	Zhao et al., 2025	Prevalence of non-alcoholic fatty liver disease in patients with inflammatory bowel disease: A systematic review and meta-analysis	26	429,550	Lin et al., 2021	Prevalence of Nonalcoholic Fatty Liver Disease in Patients With IBD: A Systematic Review and Meta-analysis	27	7,649	Prioritizes stratification by diagnostic method (imaging, elastography, biopsy) and geographic subgroups; Zhao is heavily dominated by Chinese series, limiting generalization; high risk of overlap. Comparable methodological quality.	Recency vs representativeness; diagnostic rigor; heterogeneity control; overlap of primary studies
Hepatobiliary manifestations (umbrella, multiple)	Beheshti Maal et al., 2024	Hepatobiliary manifestations in inflammatory bowel	118	1,729,128	Lin et al., 2021 (NAFLD); Barberio et al.,	NAFLD and PSC addressed by specific SR/MA	27 (NAFLD); 64 (PSC)	7,649 (NAFLD); 776,700 (PSC)	Umbrella with multiple outcomes implies high overlap with specific SRs;	Avoid multiplicity and overlap; outcome specificity

		disease: An umbrella review			2021 (PSC)				comorbidity-specific syntheses (NAFLD, PSC) preferred to avoid double counting and improve homogeneity.	
Ocular manifestations (uveitis)	Li et al., 2022	The Prevalence of Ocular Extra-Intestinal Manifestations in Adults with IBD: A Systematic Review and Meta-Analysis	21	190,941	Lin et al., 2023	Differences in the prevalence of uveitis between Crohn's disease and ulcerative colitis: A systematic review and meta-analysis	14	115,854	Provides pooled prevalences with 95% CI for IBD, CD, and UC and regional subgroups; Li primarily focuses on OR CD vs UC and mixes definitions/capture by registries, with less emphasis on prevalence pooling.	Availability of prevalence pooling; comparability; geographic subgroups
Metabolic syndrome	Janani et al., 2022	Prevalence of metabolic syndrome in patients with inflammatory bowel disease: A systematic	14	14,353	Shen et al., 2024	Prevalence of metabolic syndrome in patients with inflammatory bowel disease: Systematic review and	11	2,501	More recent SR with prespecified sensitivity analyses that reduce heterogeneity; well-defined	Recency; sensitivity analyses; consistency of definitions

		review and meta-analysis				meta-analysis with sensitivity analyses			diagnostic criteria; better control of variability.	
Anemia (iron deficiency and chronic disease)	Wilson et al., 2004	Prevalence and outcomes of anemia in inflammatory bowel disease: A systematic review	15	4,430	Filmann et al., 2014	Prevalence of anemia in inflammatory bowel diseases in European countries: Systematic review and individual patient data meta-analysis	6	2,192	More recent, with individual patient data meta-analysis and standardized WHO/ECCO definitions; Wilson reports wide ranges without formal pooling and disparate criteria.	Quality (IPD), standardization of definitions, currency
Osteoporosis	Kärnsund et al., 2020	Osteoporosis in patients with IBD: A systematic review of population-based studies	12	3,661	Marzban Abbas Abadi et al., 2025	Global prevalence of osteoporosis in patients with inflammatory bowel disease: A systematic review and meta-analysis	24	417,298	Provides overall pooled prevalence with subgroups (IBD type, sex, diagnostic method) and larger sample size; Kärnsund reports ranges without meta-analysis.	Global coverage; quantitative pooling; sample size

* Each decision is based on predefined objective criteria

Table S4. Methodological Quality Assessment (AMSTAR-2)

Author (year)	Protocol/registration	Comprehensive search	Selection/Extraction in duplicate	List of excluded studies	Description of studies	Risk of bias in primary studies	Adequacy of meta-analysis methods	Considers RoB in interpretation	Publication bias assessed	Conflicts/funding	Overall judgment (AMSTAR-2)	Notes
Lin 2021	No/NR	Yes	Yes	No/NR	Yes	Yes (NOS)	Yes (REML/random)	Partial	Yes (funnel/Egger)	Yes	Low (1 critical domain absent)	No protocol; very high heterogeneity.
Barberio 2021	No/NR	Yes	Yes	No/NR	Yes	Yes	Yes	Yes	Yes	Yes	Moderate	Covers diagnostic methods; wide heterogeneity.
Shah 2018	No/NR	Yes	Yes	No/NR	Yes	Yes	Yes	Partial	No/NR	Yes	Low	Heterogeneous SIBO methods.
Amakye 2024	No/NR	Yes	Yes	No/NR	Yes	Yes	Yes	Partial	Yes	Yes	Low-Moderate	Includes regional analyses; very high I^2.
Giri 2023	No/NR	Yes	Yes	No/NR	Yes	Yes	Yes	Partial	Yes	Yes	Low	Multiple biomarkers; high heterogeneity.

Lin 2023	No/NR	Yes	Yes	No/NR	Yes	Yes	Yes	Partial	No/NR	Yes	Low	Does not report I² for prevalence.
Karreman 2016	No/NR	Yes	No/NR	No/NR	Yes	Limited	Yes	Partial	No/NR	Yes	Low	Old and heterogeneous studies.
Filmann 2014	No/NR	Yes	Yes	No/NR	Yes	Yes	Yes	Partial	No/NR	Yes	Low	Old; high heterogeneity.
Dolatabadi 2021	No/NR	Yes	Yes	No/NR	Yes	NR/Limited	Yes	Partial	No/NR	Yes	Low	Disparate laboratory methods; small size.
Ma 2024	No/NR	Yes	Yes	No/NR	Yes	Yes	Yes	Partial	Yes	Yes	Low	Sensitivities by HRCT improve consistency.
Olfati 2023	No/NR	Yes	Yes	No/NR	Yes	Partial (NOSxc)	Yes	Partial	No/NR	Yes	Critically low	Cross-sectional studies; extreme heterogeneity.
Shi 2025	No/NR	Yes	Yes	No/NR	Yes	Yes	Yes	Partial	Partial	Yes	Low	Limited UC coverage; high heterogeneity.
Nardone 2025	No/NR	Yes	Yes	No/NR	Yes	Yes (NOS)	Yes	Partial	Yes (Egger)	Yes	Low	Includes only validated

												instruments; high heterogeneity.
Shen 2024	No/NR	Yes	Yes	No/NR	Yes	Yes	Yes	Partial	Partial	Yes	Low	Variable MS criteria.
Marzban et al. (2025)	No/NR	Partial	Yes	No	Yes	Yes	Yes	Partial	No	Yes	Critically low	Critical items unmet: protocol/registration, list of excluded studies, publication bias; duplicate extraction not reported.
Huang 2022	No/NR	Yes (multiple databases)	NR (likely, not explicit)	No/NR (PRISMA with general reasons)	Yes (detailed tables)	Yes (NOS)	Yes (random effects; meta-regression)	Partial (discusses NOS quality)	Yes (funnel/Egger/Begg)	Yes (declared)	Low	Missing protocol and citation-level list of excluded studies; extreme heterogeneity.
Kiliç 2024	No/NR	Yes (Embase/Embase	Yes (2 reviewers + third)	No/NR	Yes	Yes (JBI Prevale	Yes (random effects;	Yes (quality sensitivi	Yes (funnel, Egger,	Yes	Low (one critical limitation	Very comprehensive for

		Classic/PubMed)				nce; exclude <7/9)	JBI=9 sensitivity)	ties; meta-regression)	Begg)		without protocol/registration)	prevalences; missing registration and list of excluded studies.
Alinaghi 2019	No/NR	Yes (several databases; explicit strategy)	NR (suggests duplicate, not always explicit)	No/NR	Yes	Yes (quality assessment; NOS/criteria)	Yes (random effects; multiple strata)	Partial	Yes (funnel/Egger with reported p values)	Yes	Low	Good detail of subgroups; very high heterogeneity and variable definitions.

Legend/Notes. AMSTAR-2: tool for assessing systematic reviews of non-randomized studies; protocol/registration: existence of a priori protocol (e.g., PROSPERO); comprehensive search: multiple databases, without inappropriate language/date restrictions; selection/extraction in duplicate: two independent reviewers; list of excluded studies: listing with reasons for full-text exclusion; description of studies: key characteristics (population, exposure/diagnosis, outcomes); risk of bias in primary studies: use and reporting of appropriate tools (e.g., NOS, AHRQ, JBI); adequacy of meta-analysis: correct model, heterogeneity assessment, sensitivity/subgroup analyses; considers RoB in interpretation: integrates risk of bias when discussing findings; publication bias: formal tests (funnel, Egger/Begg) when $n \geq 10$; conflicts/funding: declaration of funding and conflicts; overall AMSTAR-2 judgment: High, Moderate, Low, or Critically low according to critical domains unmet. NR: not reported; NA: not applicable. Notes reflect exactly what was reported by the authors; when a domain is "Partial" it indicates incomplete compliance or insufficient reporting.

Table S5. Risk of Bias Assessment Using ROBIS

Author (year)	D1: Bias in eligibility criteria	D2: Bias in identification/selection	D3: Bias in data collection/appraisal	D4: Synthesis and findings	Overall ROBIS risk	Brief justification
Lin 2021	Low	Low	Low-Moderate	High (very high I^2; variability by method)	High	Extreme heterogeneity; potential bias from NAFLD diagnosis.
Barberio 2021	Low	Low	Low	High	High	Variability by diagnostic method (codes vs MRCP/ERCP/biopsy).
Shah 2018	Low	Low	Moderate	High	High	Heterogeneous SIBO definitions; different breath tests.
Amakye 2024	Low	Low	Low	High	High	Regional/CDI method diversity; I^2 99%.
Giri 2023	Low	Low	Low-Moderate	High	High	Heterogeneous markers; low prevalences with regional variability.
Lin 2023	Low	Low	Low	Moderate	Moderate	Low prevalence; mixture of registries/clinical diagnosis; I^2 NR.
Kärnsund 2020	Low	Low	Moderate	High (no MA)	High	Ranges without pooling; possible selection bias.
Karreman 2016	Moderate	Moderate	Moderate	High	High	Old studies; heterogeneous measures.
Filmann 2014	Low	Low	Low	High	High	Very high I^2 even with standardized definitions.
Dolatabadi 2021	Moderate	Moderate	Moderate-High	High	High	Non-standardized AIEC methods; small samples.
Ma 2024	Low	Low	Moderate	High	High	Variable bronchiectasis diagnosis (X-ray vs HRCT).
Olfati 2023	Moderate	Low	Moderate	High	High	Only cross-sectional; high heterogeneity.
Shi 2025	Low	Low	Moderate	High	High	Heterogeneous FI definitions; greater evidence in CD.

Nardone 2025	Low	Low	Low	High	High	Different questionnaires; high heterogeneity.
Shen 2024	Low	Low	Moderate	High	High	Diverse MS criteria between studies.
Marzban et al. (2025)	Low	High	Unclear	High	High	Limitations in search and synthesis with I^2≈100%; heterogeneous outcome definition; absence of formal RoB integration.
Huang 2022	Low	Low	Moderate (duplicate not clearly documented)	High (I^2≈100%; multiple frailty definitions)	High	Good framework and NOS, but extreme heterogeneity and variation in frailty indices; RoB integration in interpretation not always explicit.
Kiliç 2024	Low	Low	Low (duplicate extraction; JBI)	High (I^2 97–100%; mixture of sources/EIM definitions)	High	Solid methods (sensitivity, meta-regression, publication bias assessment), but very high residual heterogeneity; exclusion of non-articular/hepatic EIMs due to heterogeneity.
Alinaghi 2019	Low	Low	Moderate (duplicate detail not always explicit)	High (I^2≈98%; variable psoriasis/PsA definitions)	High	Wide coverage and publication bias tests, but great inconsistency and variability in diagnosis by region/age.

Legend/Notes. ROBIS: tool for risk of bias in systematic reviews; D1 (eligibility criteria): clarity, relevance, and applicability of inclusion/exclusion criteria; D2 (identification/selection of studies): breadth and precision of search strategy, selection without bias; D3 (data collection/appraisal): duplicate extraction, management of discrepancies, appropriate assessment of risk of bias in primary studies; D4 (synthesis and findings): adequate synthesis methods (e.g., effects model, heterogeneity control, consistency with plan), consideration of quality and heterogeneity in interpretation, exploration of publication bias. Overall ROBIS risk: Low, Some concerns, or High, determined by the pattern of domains; Brief justification: concise synthesis of reason(s) for judgment (e.g., heterogeneous diagnostic definitions, mixture of designs, dependence on ICD codes, lack of pooling). NR: not reported; NA: not applicable.
